# Characterizing the Specific Recognition of Xanthurenic Acid by GEP1 and GEP1-GCα Interactions in cGMP Signaling Pathway in Gametogenesis of Malaria Parasites

**DOI:** 10.3390/ijms24032561

**Published:** 2023-01-29

**Authors:** Cheng Zhu, Xiaoge Liang, Xu Chen, Miaomiao Liang, Jianting Zheng, Bingbing Wan, Shukun Luo

**Affiliations:** 1 School of Life Sciences and Biotechnology, Shanghai Jiao Tong University, 800 Dongchuan Road, Minhang District, Shanghai 200240, China; 2 Key Laboratory of Systems Biomedicine (Ministry of Education), Shanghai Jiao Tong University, 800 Dongchuan Road, Minhang District, Shanghai 200240, China; 3 Shanghai Center for Systems Biomedicine, Shanghai Jiao Tong University, 800 Dongchuan Road, Minhang District, Shanghai 200240, China

**Keywords:** gametogenesis, membrane protein, GEP1, GCα, AlphaFold2, protein interaction

## Abstract

Gametogenesis is an essential step for malaria parasite transmission and is activated in mosquito by signals including temperature drop, pH change, and mosquito-derived xanthurenic acid (XA). Recently, a membrane protein gametogenesis essential protein 1 (GEP1) was found to be responsible for sensing these signals and interacting with a giant guanylate cyclase α (GCα) to activate the cGMP-PKG-Ca^2+^ signaling pathway for malaria parasite gametogenesis. However, the molecular mechanisms for this process remain unclear. In this study, we used AlphaFold2 to predict the structure of GEP1 and found that it consists of a conserved N-terminal helical domain and a transmembrane domain that adopts a structure similar to that of cationic amino acid transporters. Molecular docking results showed that XA binds to GEP1 via a pocket similar to the ligand binding sites of known amino acid transporters. In addition, truncations of this N-terminal sequence significantly enhanced the expression, solubility, and stability of GEP1. In addition, we found that GEP1 interacts with GCα via its C-terminal region, which is interrupted by mutations of a few conserved residues. These findings provide further insights into the molecular mechanism for the XA recognition by GEP1 and the activation of the gametogenesis of malaria parasites through GEP1-GCα interaction.

## 1. Introduction

Malaria remains a major threat to human health, with an estimated 200 million new cases and approximately 600 thousand malaria-related deaths in 97 countries worldwide annually [1]. Artemisinin (ART) and artemisinin combination therapies (ACTs) have been widely applied in malarial treatment [1,2,3]. However, in recent years, increasing ART resistance in southeast Asia resulted in delayed parasite clearance from patients and an increased probability of treatment failure [2,3]. The parasite protein Kelch13 was found to be relevant to ART resistance, mutations of which reduced its activity and diminished the hemoglobin endocytosis critical for ART activation [4,5]. Fortunately, other antimalarial therapeutics with unconventional mechanisms are still found to be effective for malaria treatment. The blockage of glucose uptake represented an unconventional strategy for antimalarial drug development through structural analysis [6]. In the protozoan *Plasmodium falciparum*, VPS45 is crucial for host cell cytosol uptake, the inactivation of which leads to an accumulation of vesicles and inhibits the delivery of hemoglobin, resulting in arrested parasite growth [7]. Recently, several calcium-dependent protein kinases (such as *Pf*CDPK1) have also been actively pursued as targets for drug development against human malaria [8].

Male malaria parasite gametogenesis is a proliferative stage that is essential for parasite transmission from humans to the mosquito vector [9]. The gametogenesis of malaria parasites is activated in the midgut of mosquitoes following a temperature drop of approximately 5 °C. Two other stimuli have also been found to induce gamete formation, either the presence of xanthurenic acid (XA), an intermediate of tryptophan metabolism, or the pH rise from 7.4 to 8.0 [10,11]. Studies have shown that XA activates the giant membrane-anchored guanylate cyclase α (GCα) for cyclic guanosine monophosphate (cGMP) synthesis [12]. cGMP then triggers the cGMP-dependent protein kinase G (PKG) cascade, which controls the calcium signals critical for the life cycle of *Plasmodium* parasites; thus the XA-cGMP-PKG-Ca^2+^ signaling pathway was established in the gametogenesis stage [12,13,14]. In *Plasmodium yelii*, GCα was found to be responsible for the synthesis of cGMP. Recent studies showed that the membrane protein gametogenesis essential protein 1 (GEP1) interacts with GCα, which leads to the activation of the downstream cGMP signaling pathway and gametogenesis [15]. GEP1 disruption abolished the XA-stimulated synthesis of cGMP and the subsequent signaling and cellular events [15]. 

The GEP1 protein contains 905 residues, and sequence analysis showed that it has 14 transmembrane helices (TMHs). These transmembrane helices are organized into a domain similar to the 14-TMH cationic amino acid transporters SLC7A1-4 [16], and those residues locating in the ligand binding site are conserved among common malaria parasite species. However, these residues differ from known amino acid transporters, suggesting a different ligand binding ability. The tryptophan metabolite XA has been speculated to be the ligand for GEP1. Furthermore, GEP1 has a relatively conserved N-terminal region right before the transmembrane domain. GCα is a giant membrane protein with 3850 residues, including 22 transmembrane helices [15,17]. It has a unique bifunctional structure composed of an N-terminal P4-type ATPase-like domain (ALD) and a C-terminal guanylate cyclase domain (GCD) [15,17,18]. The heterodimeric GCD domain and 12 TMHs form a heterodimeric structure reminiscent of those membrane-integral adenylyl cyclases (ACs) in mammalian species [19]. However, the structure and function of GEP1, and the molecular mechanisms for its interaction with GCα, remain to be elucidated.

In this study, we set out to determine the structure of GEP1, explore its XA recognition, and understand its interaction with GCα. Large membrane proteins are exceptionally challenging to produce and are generally scarce in their native environment. In addition, recombinantly overexpressed membrane proteins are usually toxic to host cells [20,21,22]. To obtain soluble GEP1 and GCα proteins, a variety of GFP fusion protein constructs were expressed in insect cells, and the solubility was screened using the fluorescence-detection size-exclusion chromatography (FSEC) method [23,24,25,26]. Different detergents were also screened for extraction and stabilization [27,28,29,30]. We generated a putative GEP1 3D structure using AlphaFold2 and used molecular docking to understand the potential XA binding pattern and found several conserved residues that might be involved in the recognition. A slight protein conformation was observed upon pH change by circular dichroism (CD) spectroscopy. Furthermore, we have explored the interaction between GEP1 and GCα. Our data shed light on the molecular mechanisms for XA recognition by GEP1 and the interaction between GEP1 and GCα, providing further insights into the cGMP signaling pathway-dependent gametogenesis in *Plasmodium*.

## 2. Results

### 2.1. Structural Analysis of GEP1 by AlphaFold2 and the Recognition Model of XA

**Figure 1:**
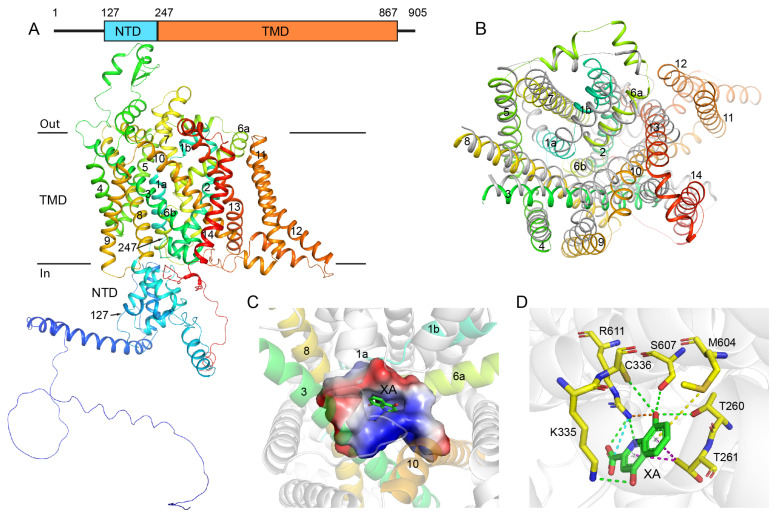
Putative structure of gametogenesis essential protein 1 (GEP1) and its binding model with xanthurenic acid (XA). (**A**) Cartoon represented structure of GEP1 predicted by AlphaFold2 colored in spectrum with N-terminus in blue and C-terminus in red. The number of the transmembrane helices and domain boundaries are indicated. (**B**) Structure superimposition of the transmembrane region between GEP1 (rainbow) and the human serotonin transporter (grey, PDB 7LI7). The extracellular and intracellular loops are hidden for the purposes of clarity. (**C**) The cartoon representation of XA docked in the ligand-binding pocket of GEP1, the binding pocket is represented as surface. (**D**) Interactions between ligand XA and protein GEP1, with XA in green sticks and residues in yellow sticks. Dashed lines indicate the interactions between the ligand and the residues of the receptor in green: hydrogen bond; yellow: pi-sulfur; purple: pi-sigma; orange: salt bridge; cyan: attractive charge.

A sequence analysis showed that GEP1 is highly conserved among *Plasmodium* species except for some N-terminal regions (Figure S1). GEP1 consists of 14 transmembrane helices (TMHs) resembling the cationic amino acid transporter subfamily SLC7A1-4 [16]. The high-throughput crystallization screening using the sitting drop vapor diffusion technique and Cryo-EM trials were not successful. Thus, we performed de novo structure prediction using recently available AlphaFold2. The predicted GEP1 model has relatively high average confidence pLDDT scores, except for some N and C-terminal regions (Figure S2). The overall structure is composed of a 14-TMH transmembrane domain (AA 247–867), an intracellular N-terminal helical domain (AA 127–246), a C-terminal tail (AA 868–905), and a few extracellular (EL) and intracellular (IL) loops (Figure 1A and Figure S3).

The transmembrane domain formed by 14 TMHs has been shown to have a high structural similarity with amino acid transporters through a structural search on the DALI server against the full PDB database (http://ekhidna2.biocenter.helsinki.fi/dali/, accessed on 10 December 2022). The search results contain transporters mostly with 12 TMHs rather than SLC7A1-4 due to the lack of structures for this subfamily (Table S1). At the top of the list are a few structures of the serotonin transporter. The hit with the highest Z-score is the human serotonin transporter SLC6A4 (PDB 7LI7), with only 12 TMHs [31]. However, these 12 transmembrane helices superimpose well with GEP1 (Figure 1B), although the residues in the ligand binding pocket are distinct. GEP1 also contains a soluble N-terminal helical domain (NTD, AA 127–246) (Figure 1A), which packs tightly against the transmembrane domain via two loops (TMH2-3 and TMH6-7). This packing is mainly mediated by hydrophobic residues. Except for the region AA 165–195, the NTD domain is relatively conserved in GEP1 orthologs in the *Plasmodium* species (Figure S1). A search on the DALI server for similar structures resulted in no similar structures, with only a few entries with a Z-score lower than 4 (Table S2). 

Intriguingly, serotonin (5-hydroxytryptamine or 5-HT) is also an intermediate in tryptophan metabolism, similarly to XA [32]. In order to find out whether GEP1 recognizes the XA molecule in its potential ligand binding pocket, we carried out molecular docking using Discovery Studio [33]. The top-ranked model showed that XA fitted in the pocket between the TMHs 1, 3, 6, 8 and 10 (Figure 1C), a common ligand binding pocket within SLC6A2-4 subfamily transporters [31,34,35]. This putative XA recognition is mediated by a few hydrogen bonds and other types of interactions with a few conserved residues, including R611, T260, T261, K335, C336, M604, and S607 of GEP1 (Figure 1D and Figure S4). However, those residues in the pocket, the orientation of XA, and the binding patern are different from that of the serotonin transporter.

### 2.2. Expression of GEP1 in Insect Cells

**Figure 2:**
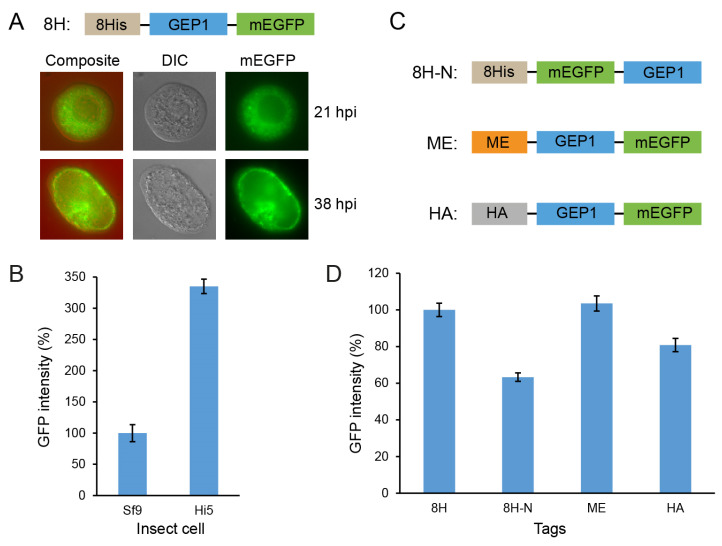
Localization and expression of GEP1 in insect cells. (**A**) Localization of mEGFP-labeled GEP1 in Hi5 infected cells. Differential interference contrast (DIC), monomeric enhanced green fluorescent protein (mEGFP). Cells were examined by inverted fluorescence microscopy. (**B**) Expression levels of GEP1 in both Sf9 and Hi5 insect cell lines. (**C**,**D**) Constructs with different tags on GEP1 and protein expression levels detected by GFP intensity. ME: Honey bee melittin signal peptide; HA: haemagglutinin signal peptide. The GFP intensity in the supernatant was measured after extraction with detergent, detected by a microplate reader (excitation 485 nm, emission 528 nm).

To further characterize GEP1, we made four constructs and expressed them using a baculovirus-insect cell system. To check the cellular localization, we fused GEP1 with a C-terminal mEGFP tag and visualized the cell by inverted fluorescence microscopy. GEP1 is scattered in the cytoplasm instead of the cell membrane of the Hi5 insect cell at 21 h post-infection (hpi). At 38 hpi, most of the green fluorescence concentrated to the narrowed cytoplasm due to the enlargement of the infected nucleus (Figure 2A). Through GFP fluorescence, we found that the protein expression level was three times higher in the Hi5 cell line than in the Sf9 cell line (Figure 2B); hence, the Hi5 cell line was selected for protein expression in this study. To improve the expression, we have generated three constructs with different tags and fusion positions (Figure 2C). We found that N-terminal 8× His and mEGFP tag GEP1 had a lower expression than the C-terminal mEGFP-tagged GEP1 (Figure 2D). The addition of ME (honey bee melittin sequence) or the HA (haemagglutinin) signal peptides, commonly used to facilitate membrane protein expression and membrane localization, provides no improvement in the expression. We have also explored the expression time via a time course assay and found that 54 hpi gives the best expression (Figure S5). Thus, the C-terminal mEGFP fusion construct and the 54 hpi expression time were used for expression in the following study.

### 2.3. N-terminal Truncation Facilitates Protein Expression and Solubility

**Figure 3:**
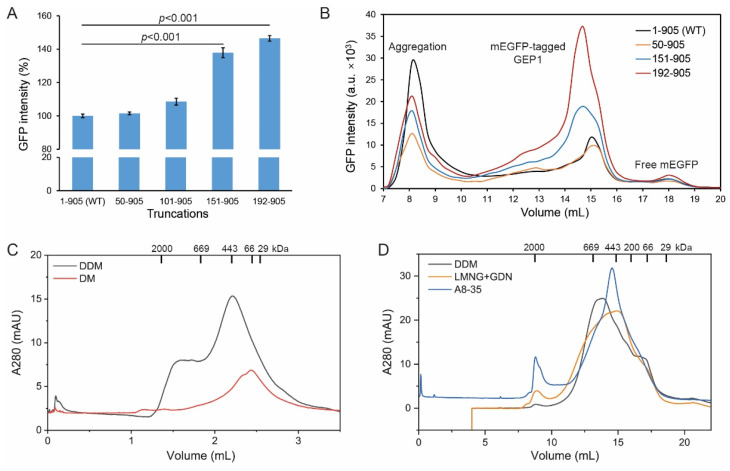
N-terminal truncation and trapping in A8-35 increased the expression level and stability of GEP1. (**A**) Expression level of N-terminal truncation on GEP1 with mEGFP tag; data were analyzed using a two-tailed unpaired Student’s *t*-test. (**B**) Fluorescence-detection size-exclusion chromatography (FSEC) traces of crude detergent extract of GEP1 truncations with mEGFP tag. (**C**) Size exclusion chromatography (SEC) profiles of GEP1^151-905^ in detergent DDM and DM. The dispersity was determined on a Superose 6 Increase 5/150 GL column. (**D**) SEC profiles of GEP1^151-905^ solubilized in detergents or trapped in amphipol A8-35. The dispersity was determined on a Superose 6 Increase 10/300 GL column.

Unlike other amino acid transporters, GEP1 harbors an extra 246 residues at its N-terminus upstream to the TMH-1. To improve the expression and solubility, we generated a series of N-terminal truncations and compared their expression level via GFP intensity. The results showed that the GEP1^50-905^ and GEP1^101-905^ truncations have similar expression to the wild type (WT), while the GEP1^151-905^ and GEP1^192-905^ are significantly higher (*p* < 0.001) (Figure 3A). We proceeded to extract these truncation mutants using the detergent n-dodecyl-β-d-maltoside (DDM) and assessed their behavior in solution using fluorescence-detection size-exclusion chromatography (FSEC). The WT extraction has a large aggregation peak and much lower soluble fractions, with a soluble/aggregate ratio of 0.48. The truncation mutants GEP1^151-905^ and GEP1^192-905^ have much higher soluble fractions with soluble/aggregate ratios of 0.95 and 1.39, respectively (Figure 3B). The DDM and cholesteryl hemisuccinate (CHS) detergent mixture is commonly used to solubilize and stabilize membrane proteins, and we found that 2%/0.4% DDM/CHS has better extraction capability for GEP1^192-905^ (Figure S6) than DDM only. These results indicated that specific N-terminal truncations are beneficial for protein expression and solubility, and adding CHS promoted the efficiency of the extraction.

### 2.4. Protein Purification and Trapping in Amphipol Enhances Homogeneity

GEP1 with an mEGFP tag was originally used for construct screening and two-step purification using amylose resin and mEGFP-nanobody, but the yield and purity of protein did not meet the requirements. For the purposes of purification for structural and biochemical characterization, we replaced the mEGFP tag with Strep-tag, which is shorter and has higher specificity. With this tag, we have obtained protein for the truncation mutants GEP1^151-905^ and GEP1^192-905^ with relatively high purity (Figure S7). However, the purification failed for full-length GEP1 due to its lower solubility. We have also tested detergents other than DDM, such as n-decyl-β-d-maltoside (DM) [27], lauryl maltose neopentyl glycol (LMNG), and glyco-diosgenin (GDN) [36,37,38]. The SEC profiles showed that GEP1^151-905^ is less soluble in DM, with a smaller peak and longer retention time compared with DDM (Figure 3C). The detergent mixture of LMNG and GDN performed similarly to the DDM (Figure 3D). An SDS-PAGE analysis confirmed that the SEC elution peak contains our target protein (Figures S8 and S9). We have also tested these solution samples via negative stain electron microscopy, and the images showed that particles were uniformly dispersed with a small amount of aggregation (Figure S10). In addition, we obtained stable and monodisperse GEP1^151-905^ protein trapped in the amphipol A8-35, a detergent-free reagent that can mimic the native environment (Figure 3D and Figure S11), facilitating the subsequent study of GEP1 properties.

### 2.5. CD Spectroscopy of GEP1 Protein at Different pH and Temperatures

**Figure 4:**
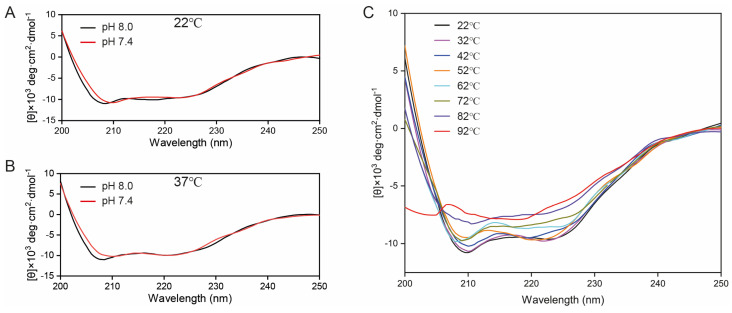
Circular dichroism (CD) spectra of GEP1^151-905^ in different pH and temperatures. (**A**) Overlay of CD spectra in pH 7.4 and 8.0 at 22 °C. (**B**) Overlay of CD spectra in pH 7.4 and 8.0 at 37 °C. (**C**) Temperature-dependent CD spectra in buffer 5 mM HEPES (pH 7.4) and 150 mM NaCl; the temperature range is from 22 °C to 92 °C and is varied in steps of 10 °C.

An analysis of GEP1^151-905^ by circular dichroism (CD) spectroscopy revealed that the sample exhibits an α-helical conformation [39] which has two negative bands at 222 nm and 208 nm (Figure 4A–C). When the pH was increased from 7.4 to 8.0, the negative ellipticity in the 208 nm was enhanced at both 22 °C and 37 °C (Figure 4A–B) due to a decrease in the 222/208 nm ratio, indicating the partial unpacking of coiled helices [40,41]. As the pH increase from 7.4 to 8.0 is an inducer for gametogenesis [10,15], this signaling process might involve the potential unpacking of coiled helices. However, we did not observe significant conformational change, as it had similar characteristic negative bands at both 22 °C and 32 °C. As the temperature rose from 42 °C to 72 °C, the negative ellipticity was decreased gradually both at 208 nm and 222 nm. The characteristic signal of α-helices disappeared as the temperature went beyond 82 °C (Figure 4C and Figure S12).

### 2.6. GEP1 Interacts with GCα via Its C-terminal Domain

**Figure 5:**
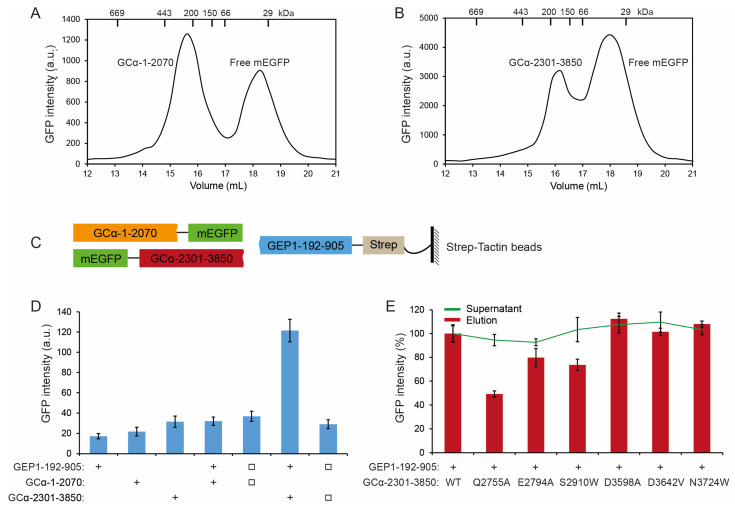
Expression of guanylate cyclase α (GCα) and its interaction with GEP1^192-905^. (**A**,**B**) FSEC profiles of GCα^1-2070^ with C-terminal mEGFP and GCα^2301-3850^ with N-terminal mEGFP. Samples were run on a Superose 6 Increase 10/300 GL column using buffer (5 mM HEPES pH 7.6, 150 mM NaCl, 0.03% DDM, 2 mM β-ME). GFP intensity was detected by a microplate reader (excitation 485 nm, emission 528 nm). (**C**) Schematic diagram for probing GEP1-GCα interaction. (**D**) mEGFP tagged N-terminal half (GCα^1-2070^) and C-terminal half (GCα^2301-3850^) were used for a pull-down assay with Strep-tagged GEP1^192-905^ using Strep-Tactin beads. ‘+’ indicates co-expression, and ‘□’ indicates co-purification of proteins expressed separately. (**E**) Mutations on GCα^2301-3850^ were used for a pull-down assay with Strep-tagged GEP1^192-905^ using Strep-Tactin beads. A green line shows the GFP intensity in the supernatant, indicating the expression level for each mutant.

In *Plasmodium* species, GCα is indispensable as a cGMP synthesizing enzyme in gametogenesis. To understand this signaling process, we set out to express GCα and test its interaction with GEP1. However, the extremely large molecular weight and the structural complexity of GCα made its cloning, expression, and purification challenging. A recent study showed that the attempt to express the GCD, including the 12 TMHs of *Tg*ATPase_P_-GC, was unsuccessful [42]. We have cloned the full-length GCα and fused it with a C-terminal mEGFP tag for insect cell expression. A weak GFP fluorescent was observed in the cell, but no soluble peak appeared on the FSEC profile after DDM extraction. We then made several constructs that cover either the ALD or GCD domain, including GCα^1-2070^ (GCα-N) and GCα^2301-3850^ (GCα-C) tagged with mEGFP. Our FSEC profile showed that GCα^1-2070^ with a C-terminal mEGFP tag expressed well and was soluble in 2%/0.4% DDM/CHS (Figure 5A). For GCD, we obtained one soluble fragment containing GCα^2301-3850^ tagged with an mEGFP at its N-terminus (Figure 5B).

Furthermore, we added Strep-tag to the N or C-terminus of GCα^1-2070^ and GCα^2301-3850^. However, the expression levels of these two fragments were relatively low, and the purification using the Strep-Tactin beads failed. Since GEP1 has been proposed to interact indirectly with GCα in vivo by co-immunoprecipitation and co-localization analysis [15], we wanted to verify this interaction by pull-down assay using recombinant mEGFP tagged GCα fragments and Strep-tagged GEP1 protein (Figure 5C). We did not observe a significant change in GFP intensity for GCα^1-2070^ and GEP1^192-905^ when they were co-expressed or co-purified compared with either expressed alone. However, GCα^2301-3850^ showed a much higher GFP intensity when co-expressed but not co-purified with GEP1^192-905^, which might be due to the complex formation that improves stability and expression (Figure 5D). Thus, we hypothesize that the C-terminal half of GCα (GCα^2301-3850^ or GCα-C) containing the GCD domain interacts directly with GEP1.

**Figure 6:**
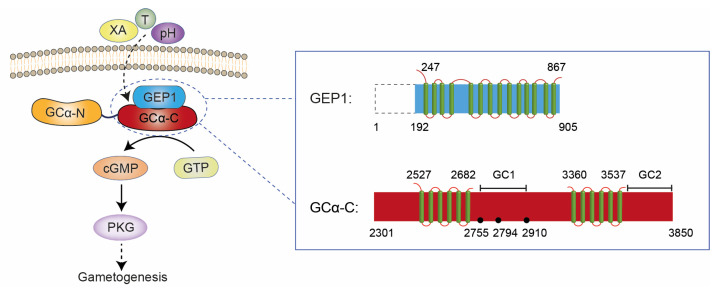
A working model of GEP1/GCα-dependent cGMP signaling pathway essential for gametogenesis. Domain organization and mutation sites are shown on the right. XA: xanthurenic acid; T: temperature; cGMP: cyclic guanosine monophosphate; GTP: guanosine triphosphate; PKG: protein kinase G; GC1: guanylate cyclase 1 domain; GC2: guanylate cyclase 2 domain. Green bars indicate TMHs and black dots indicate the mutation sites.

To confirm this observation, we have made six point-mutations of highly conserved residues on GCα-C based on sequence alignment among those *Plasmodium* species. The difference in GFP intensity in the supernatant was within 10%, suggesting that the mutation of GCα had no significant impact on the expression of GCα (Figure 5E). However, the GFP intensity in the pull-down eluates exhibited some difference. Notably, the GFP signal dropped significantly in the three mutations of the predicted highly conserved and exposed residues Q2755, E2794, and S2910 (Figure 5E and Figure S13), especially in Q2755A, where the GFP intensity further reduced to 49.2% of WT, indicating that residue Q2755 may be involved in the interaction of GEP1 and GCα-C (Figure 6).

## 3. Discussion

Gametogenesis is essential for malaria parasite transmission, and the XA-cGMP-PKG-Ca^2+^ signaling pathway has already been established [12,13,14]. Membrane-anchored guanylyl cyclase GCα has been revealed to be responsible for cGMP synthesis during gametogenesis, and the activity and regulation of GCα require another membrane protein GEP1 and upstream signals, including temperature change, pH change, and a tryptophan metabolism intermediate xanthurenic acid (XA) [15]. However, the structure and function of GEP1 and its interaction with GCα are largely unknown. In this study, we used bioinformatic methods to gain structural information and the XA binding property of GEP1. Through de novo structure prediction by AlphaFold2, we found that GEP1 is composed of an N-terminal conserved domain and a transmembrane domain with 14 TMHs reminiscent of that of the cationic amino acid transporter subfamily SLC7A1-4 [16]. Molecular docking results showed that XA is recognized in the canonical ligand binding pocket of GEP1 surrounded by a few residues conserved within the *Plasmodium* species. We have also expressed a few GEP1 truncations in the insect cell and explored membrane extraction and purification strategies. We have also successfully expressed several truncations for the giant membrane protein GCα in insect cells and found that its C-terminal GCD domain interacts with GEP1. 

We have observed a slight conformational change of GEP1 upon pH shift by circular dichroism spectrometry, indicating that GEP1 might change its conformation for downstream cGMP signaling. pH change-induced conformational change is common in the membrane transporter family proteins. For example, the amino acid-polyamine-organocation transporter GadC was reported to be inactive and conformationally homogeneous at neutral pH. However, when it changes into acidic pH, isomerization between two conformations occurs, which leads to the detachment of its C-terminus from the transmembrane domain and a higher structural disorder [43].

Furthermore, our molecular docking results showed that XA binds to the canonical ligand-binding pocket in GEP1 through a variety of specific interactions with conserved residues. We hypothesize that XA binding causes a conformational change and alters its interaction with GCα, which regulates the enzyme activity for the cGMP synthesis. Numerous structural studies regarding transporters revealed that ligand recognition is often accompanied by conformational changes. Human serotonin transporter was reported to assume conformational changes upon binding to serotonin or other inhibitors, especially the transmembrane helices TM1 and TM6. These changes locked the transporter in an outward-open conformation by restricting transporter isomerization into an occluded and inward-open state [44]. This phenomenon is similar in dopamine transporters and leucine transporters [34,35,45,46]. 

Distinct from most amino acid transporters, GEP1 also contains a conserved N-terminal helical domain and some less conserved flexible sequences on both termini, the functions of which are unknown. Based on the AlphaFold2-predicted structure, the N-terminal domain packs tightly via hydrophobic interaction with the transmembrane domain. The linker connecting to the transmembrane domain is short and is involved in the packing, which might restrict the interdomain movement. No similar structure was found for this N-terminal domain based on our DALI search results. We propose that this domain acts as a structure stabilizer for the transmembrane domain, which helps lock GEP1 in a specific conformation until the ligand XA binds. It could also act as a gate regulator for XA transporting activity. Some intracellular regions are found to regulate the transporting activity of the transmembrane domain, as exemplified by the *Drosophila melanogaster* dopamine transporter (DAT), which has a C-terminal helix as a latch that caps the intracellular gate [35].

Our results also confirmed that GEP1 interacts with GCα via its GCD domain. We hypothesize that, upon XA binding and pH change, GEP1 undergoes a conformational change and interacts with GCα to activate cGMP production, which then activates PKG and downstream signaling events during gametogenesis (Figure 6). GCs with ALD and GCD domains were found in many protozoan species [47,48]. In *Toxoplasma gondii*, the model apicomplexan, *Tg*GC possesses an ALD domain fused to a C-terminal heterodimeric GCD domain. Complementation experiments revealed that the ALD domain also plays an essential role for maximum GC function [18,49]. Intriguingly, two co-factor proteins, CDC50.1 and UGO, were reported to interact with *Tg*GC via its ALD and GCD domains, respectively. UGO is a membrane protein with 12 TMHs and intracellular regions, and a study showed that it might interact with the GCD domain as a chaperone to regulate its guanylate cyclase activity essential for the downstream signaling pathway [49]. GEP1 might adopt a similar manner to regulate guanylate cyclase activity, although it shares no structural similarity with UGO based on AlphaFold2 prediction. Our results also showed that mutations on the GCD domain impaired the affinity with GEP1, suggesting that the interaction might be mediated via the intracellular domains of these two proteins. However, further studies will be needed to confirm this interaction and to determine how the catalytic activity of the GCD domain is regulated.

## 4. Materials and Methods

### 4.1. Regents

DDM, CHS, LMNG, GDN and A8-35 were purchased from Anatrace (Maumee, OH, USA). ESF 921 Insect Cell Culture Medium was obtained from Expression Systems (Davis, CA, USA). Size exclusion chromatography columns (Superose 6 Increase 10/300 GL and Superose 6 Increase 5/150 GL) were purchased from Cytiva (Marlborough, MA, USA). Strep-Tactin^®^XT beads and BioLock were purchased from IBA Life Sciences (Göttingen, Germany).

### 4.2. De Novo Structure Prediction Using AlphaFold2 and Molecular Docking Using Discovery Studio

The protein structure of GEP1 was predicted using AlphaFold2 [50] through the ColabFold project (https://github.com/sokrypton/ColabFold, accessed on 1 September 2022) [51]. Multiple sequence alignment for prediction was generated against both the UniRef and Environmental databases using both paired and unpaired sequences by the MMseqs2 method. Predictions were run over three cycles, and the five models generated were relaxed using Amber and ranked by PAE and pTMscore [52]. Molecular docking was performed using Discovery Studio 4.0. Ligand XA was prepared by the addition of hydrogen atoms and energy minimization. Protein GEP1 as a receptor was prepared by the addition of hydrogen atoms and optimization using a CHARMM force field [53]. A LibDock module was used for docking, and the pose with the highest score was chosen as the binding model. Pymol was used for figure preparation (https://pymol.org/2/, accessed on 1 January 2022).

### 4.3. Construct Design

GEP1 (XP_022812386) and GCα (XP_724759) from *Plasmodium yoelii* were cloned into the baculovirus shuttle plasmid pKL [54] using Transfer-PCR techniques [55]. The ME signal peptide (MKFLVNVALVFMVVYISYIYAD) and HA signal peptide (MKTIIALSYIFCLVFA) were added to N-terminus of GEP1 by Transfer-PCR. N-terminal truncations of GEP1 and mutations of GCα-C were constructed using the QuickChange^TM^ method. PCR amplification was performed using PrimeSTAR DNA polymerase (Takara, Okinawa, Japan). After eliminating the template with DpnI (Takara, Okinawa, Japan), the PCR product was transformed into solid medium containing kanamycin resistance for screening. Primers used to generate constructs with different tags are listed in Table S3, primers for N-terminal truncations of GEP1 are listed in Table S4, and primers for mutants of GCα-C are listed in Table S5. All constructs were verified by sequencing.

### 4.4. Protein Expression

GEP1 was expressed according to the Bac-to-Bac^®^ baculovirus expression system (ThermoFisher, Waltham, MA, USA). Constructs were transformed into DH10Bac competent cells and recombinant bacmid DNA were prepared before being transfected into Sf9 cells. A P1 virus was produced and the P2 and P3 virus were amplified for large expression. Hi5 cells were cultured in an ESF 921 Insect Cell Culture Medium at 27 °C and 120 rpm, and infected at the density of 2.0 × 10^6^ cell/mL. The expression level was frequently checked by GFP intensity using inverted fluorescence microscopy. Finally, cells were collected at 54 hpi by centrifugation at 800× *g*. The cell pellets were flash frozen by liquid nitrogen and stored at −80 °C.

### 4.5. Observation Using Inverted Fluorescence Microscopy

Cells were placed between the slide and cover glass, and then a drop of oil was added on the cover glass prepared for observation. The differential interference contrast (DIC) and fluorescent images were recorded by an Olympus IX83 inverted fluorescence microscope equipped with a Hamamatsu Orca Flash4.0 LT camera and a Lumencor Spectra X six-channel light source. Magnification: eyepiece lens 10×, objective lens 100×. The images were adjusted for optimum brightness and contract using ImageJ software (https://imagej.nih.gov/ij/, accessed on 10 March 2021).

### 4.6. Detection of GFP Intensity and FSEC

The harvested cells were resuspended in a lysis buffer containing 50 mM Tris-HCl pH 8.0, 150 mM NaCl, 5% glycerol, 1× protease inhibitor cocktail (Apexbio, Houston, TX, USA), 5 mM β-mercaptoethanol (β-ME), and 1 mM phenylmethylsulfonyl fluoride (PMSF). The cell suspension was lysed by sonication and protein was extracted by 1% (*w*/*v*) DDM at 4 °C for 2 h. Cell debris was spun down by high-speed centrifugation (50,000× *g*) at 4 °C for 1 h. Expression levels by GFP intensity were measured by a microplate reader (excitation 485 nm, emission 528 nm). All experiments were conducted in triplicate. A two-tailed unpaired Student’s *t*-test was applied for comparison between truncations and WT, *p* < 0.001 indicated statistical significance.

For FSEC detection, the supernatant was loaded to a Superose 6 increase 10/300 GL column equilibrated with a running buffer (5 mM HEPES pH 7.6, 150 mM NaCl, 0.03% DDM, 2 mM β-ME). The flow rate was 0.5 mL/min and fractions were collected as 0.3 mL per tube. The fluorescence intensity for each fraction was measured by microplate reader (excitation 485 nm, emission 528 nm).

### 4.7. Pull-Down Experiment

The harvested cells were resuspended in lysis buffer (50 mM Tris-HCl pH 8.0, 150 mM NaCl, 5% glycerol, 1× protease inhibitor cocktail, 5 mM β-ME, and 1 mM PMSF). Cells were lysed by sonication and extracted using a lysis buffer with 2%/0.4% DDM/CHS at 4 °C for 2 h. Cell debris was spun down by high-speed centrifugation (50,000× *g*) at 4 °C for 1 h. The supernatant was then incubated with Strep-Tactin beads supplemented with 0.5 mM EDTA at 4 °C for 20 min. The beads were washed five times with buffer (50 mM Tris-HCl pH 8.0, 150 mM NaCl, 0.06%/0.012% DDM/CHS, 0.5 mM EDTA). Finally, protein was eluted using a buffer (50 mM biotin, 50 mM Tris-HCl pH 8.0, 150 mM NaCl, 0.06%/0.012% DDM/CHS, 0.5 mM EDTA).

### 4.8. Protein Purification

The harvested cells were resuspended in a hypotonic buffer (20 mM Tris-HCl pH 8.0, 5 mM 2-ME) and incubated at 4 °C for 30 min before centrifugation at 50,000× *g* for 30 min. The pellet containing cell membrane components was collected and resuspended in an extraction buffer (50 mM Tris-HCl pH 8.0, 150 mM NaCl, 5% glycerol, 1× protease inhibitor cocktail, 5 mM β-ME, 1 mM PMSF). Membrane suspension was further sonicated and extracted by 2%/0.4% DDM/CHS at 4 °C for 2 h. The extraction solution was spun down again by high-speed centrifugation (50,000× *g*) at 4 °C for 1 h. Biolock (1 mL/L culture) was added to the supernatant and incubated at 4 °C for 20 min. Strep-Tactin beads and 0.5 mM EDTA were added and incubated at 4 °C for another 20 min. The beads were then transferred to a gravity column and washed with a buffer (50 mM Tris-HCl pH 8.0, 150 mM NaCl, 0.06%/0.012% DDM/CHS, 0.5 mM EDTA) and finally eluted with a buffer (50 mM biotin, 50 mM Tris-HCl pH 8.0, 150 mM NaCl, 0.06%/0.012% DDM/CHS, 0.5 mM EDTA). The Strep-purified GEP1 protein was loaded onto a Superose 6 Increase 5/150 or 10/300 GL column using a buffer plus various detergents to assess their dispersity.

### 4.9. Negative Stain Electron Microscopy

The morphology of membrane protein GEP1 was analyzed by taking transmission electron microscopy (TEM) photographs using a Talos F200C G2 (ThermoFisher, Waltham, MA, USA), operating at 200 kV. The samples were prepared by first placing a drop of protein solution (4 μL) onto a carbon-coated 200-mesh copper grid (Zhongjingkeyi, Beijing, China). After one minute, the excess solution was wicked off by filter paper. The grid containing protein was gently washed once with ultra-pure water. The staining agent uranyl acetate (2% *w*/*v*; 4 μL) was then placed on the grid, where it stayed for an extra minute, and the excess solution was wicked off by filter paper. The previous step of staining was repeated, and the grid was left to dry under ambient conditions.

### 4.10. Trapping in Amphipol A8-35

Strep-purified GEP1 protein was mixed with amphipol A8-35 at a protein: amphipol ratio of 1:5 (*w*/*w*), then agitated gently at 4 °C for 1 h protected from light. The detergent was removed by 15 mg/mL Bio-Beads SM-2 (Bio-rad, Hercules, CA, USA) at 4 °C overnight protected from light. The bio-beads were then removed over a disposable polyprep column, and the eluent was cleared by centrifugation before further purification by a Superose 6 increase 10/300 GL column in a running buffer (5 mM HEPES pH 7.6, 150 mM NaCl).

### 4.11. CD Spectroscopy Analysis

CD spectra were recorded on a J-1500 Circular Dichroism Spectrophotometer equipped with a thermostatted cell holder. Experiments were performed with a 1 mm path length cell over a 200–250 nm wavelength range at various temperatures, 0.5 nm data pitch, and a bandwidth of 2 nm. The protein concentration was 0.12 mg/mL, and experiments were repeated three times to reduce noise. 

### 4.12. Detection of Interaction between GEP1 and GCα

GEP1 constructs were labeled with Strep-tag, and GCα constructs were labeled with mEGFP tag. In the co-expression experiment, the same amount of virus was added to infect Hi5 cells. In the co-purification experiment, each protein was expressed separately and the extracted supernatant was mixed before incubation with beads. The elution was obtained according to the method of a pull-down experiment. The GFP intensity was measured by a microplate reader (excitation 485 nm, emission 528 nm). All experiments were conducted in triplicate.

## 5. Conclusions

In summary, we predicted and analyzed the structure of GEP1 and the binding mode of GEP1-XA using computational methods. GEP1 and two domains of GCα were expressed in insect cells, and the solubility and stability of GEP1 were improved by N-terminal truncation and the optimization of detergents. We further confirmed that GEP1 interacts with GCα via its C-terminal region, and mutations on a few conserved residues could impair this interaction, especially the Q2755A. Our findings provided further insights into the molecular mechanism for the activation of gametogenesis of malaria parasites through the cGMP signaling pathway involving XA, GEP1, and GCα.

## Data Availability

Not applicable.

## Supplementary Materials

The following supporting information can be downloaded at: https://www.mdpi.com/article/10.3390/ijms24032561/s1.
