# Supplementary material for: Characterizing the Specific Recognition of Xanthurenic Acid by GEP1 and GEP1-GCα Interactions in cGMP Signaling Pathway in Gametogenesis of Malaria Parasites
Source: Int J Mol Sci. 2023 Jan 29;24(3):2561. doi: 10.3390/ijms24032561 (PMC9916804; doi:10.3390/ijms24032561)
Supplement: Supplementary file 1 [file ijms-24-02561-s001.zip › ijms-2181023-supplementary.pdf]

# Supplementary Materials

## Characterizing the Specific Recognition of Xanthurenic Acid by GEP1 and GEP1-GCα Interactions in cGMP Signaling Pathway in Gametogenesis of Malaria Parasites

Cheng Zhu [1], Xiaoge Liang [1], Xu Chen [1], Miaomiao Liang [1], Jianting Zheng [1], Bingbing Wan [2,3], Shukun Luo [1,*]

[1] School of Life Sciences and Biotechnology, Shanghai Jiao Tong University, 800 Dongchuan Road, Minhang District, Shanghai, 200240, China

[2] Key Laboratory of Systems Biomedicine (Ministry of Education), Shanghai Jiao Tong University, 800 Dongchuan Road, Minhang District, Shanghai 200240, China

[3] Shanghai Center for Systems Biomedicine, Shanghai Jiao Tong University, 800 Dongchuan Road, Minhang District, Shanghai 200240, China

* Correspondence: shukunluo@sjtu.edu.cn

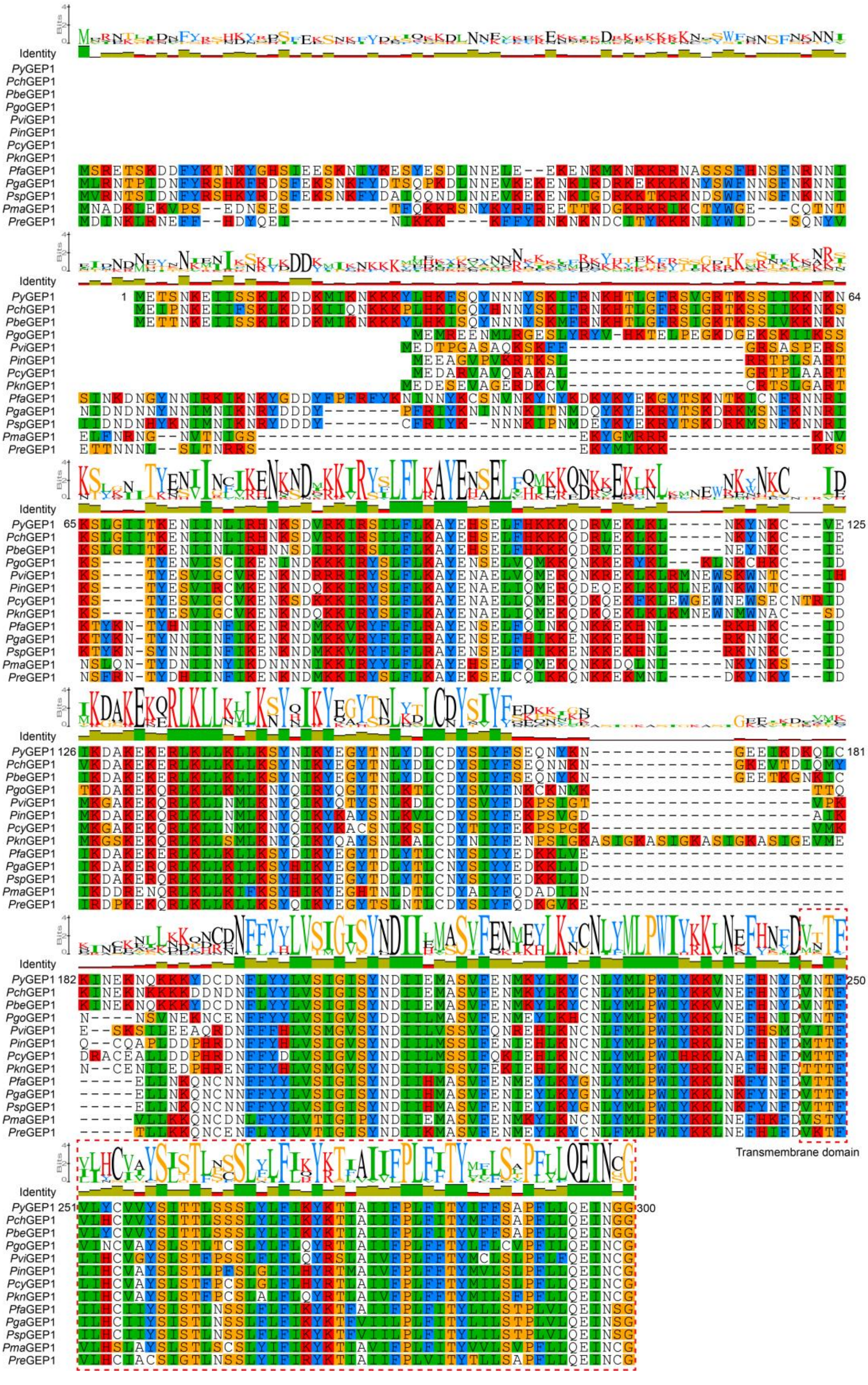

Figure S1. Multiple sequence alignment of the first 300 residues of GEP1 from 13 species of *Plasmodium*. The analysis was conducted with software geneious (http://www.geneious.com). Species used for the alignment were *Py*: *Plasmodium yelii*; *Pch*: *Plasmodium chabaudi chabaudi*; *Pbe*: *Plasmodium berghei ANKA*; *Pgo*: *Plasmodium gonderi*; *Pvi*: *Plasmodium vivax*; *Pin*:

*Plasmodium inui San Antonio 1*; *Pcy*: *Plasmodium cynomolgi B*; *Pkn*: *Plasmodium knowlesi strain H*; *Pfa*: *Plasmodium falciparum 3D7*; *Pga*: *Plasmodium gaboni*; *Psp*: *Plasmodium sp. gorilla clade G2*; *Pma*: *Plasmodium malariae*; *Pre*: *Plasmodium relictum*.

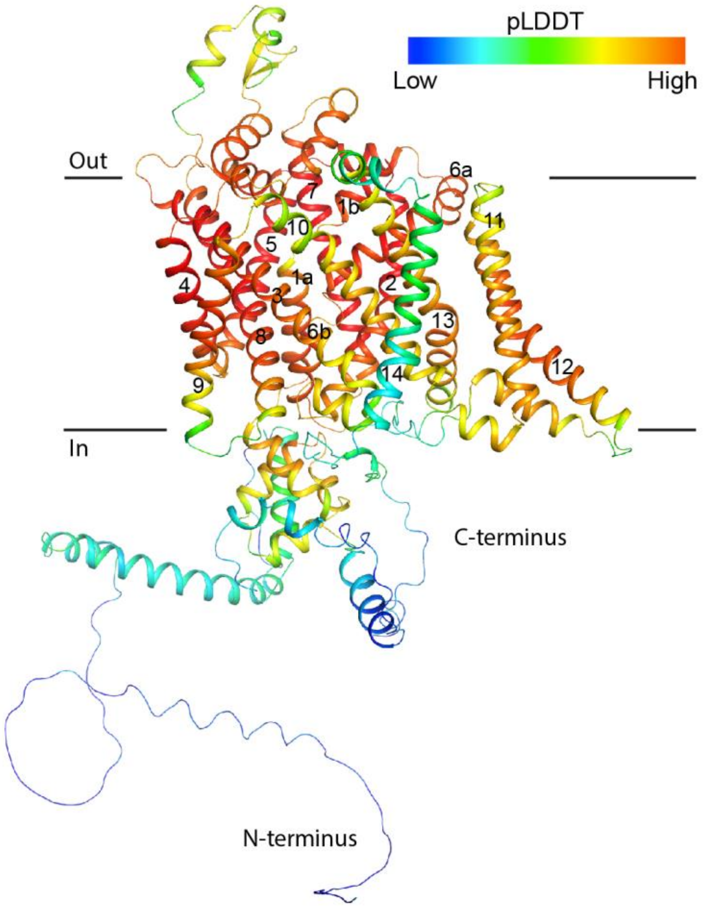


Figure S2. *De novo* structure prediction using AlphaFold2 for GEP1 protein. Model is colored by pLDDT value.

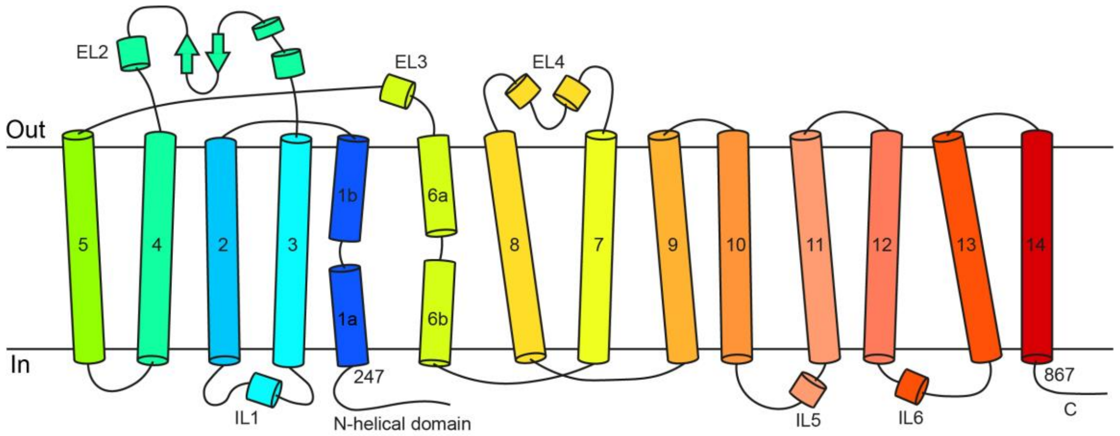


Figure S3. Membrane topology of GEP1 protein. This schematic topology representation is based on the 3D structure predicted by AlphaFold2.

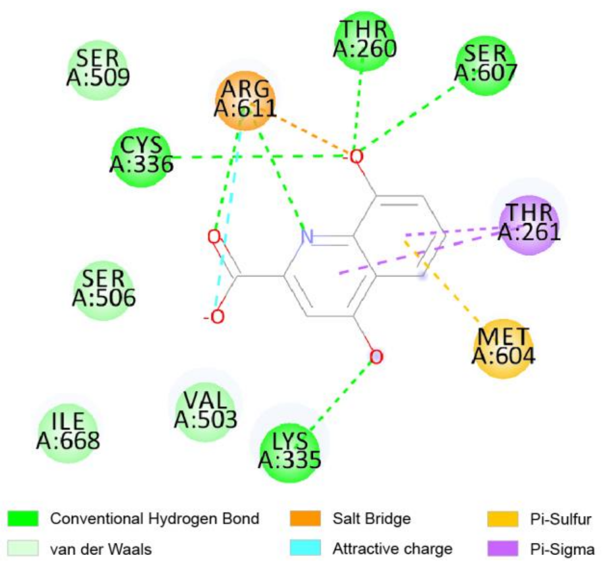


Figure S4. 2D ligand interaction diagram between XA and GEP1.

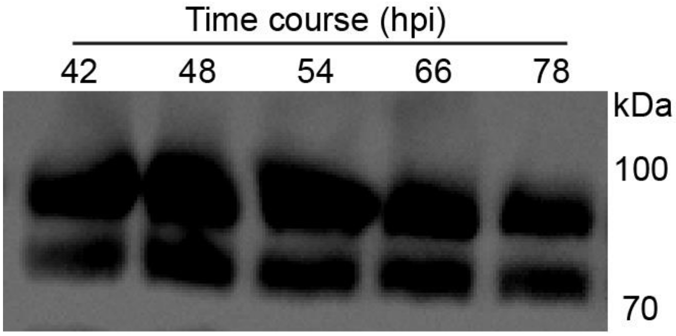


Figure S5. Comparison of expression level of mEGFP fused GEP1$^{192-905}$ with hours post-infection (hpi) by western blot, anti-rabbit GFP-tag antibody was used.

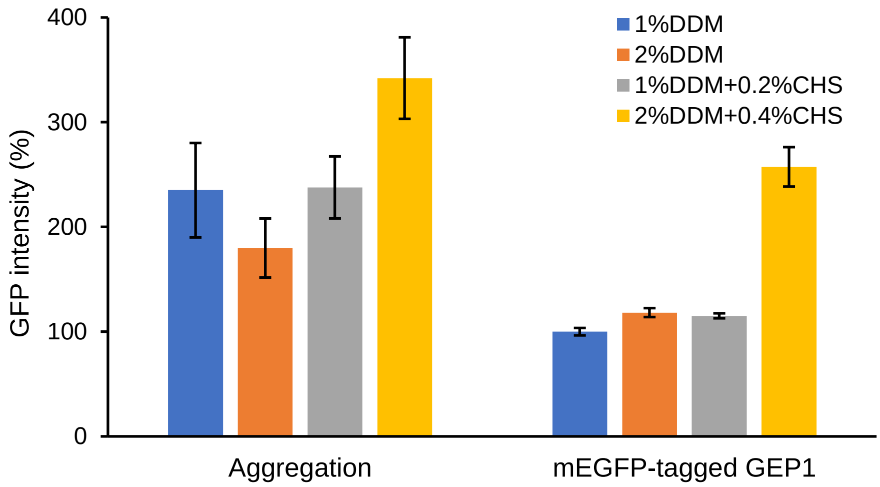

Figure S6. Optimization of detergents for the extraction of membrane protein GEP1.

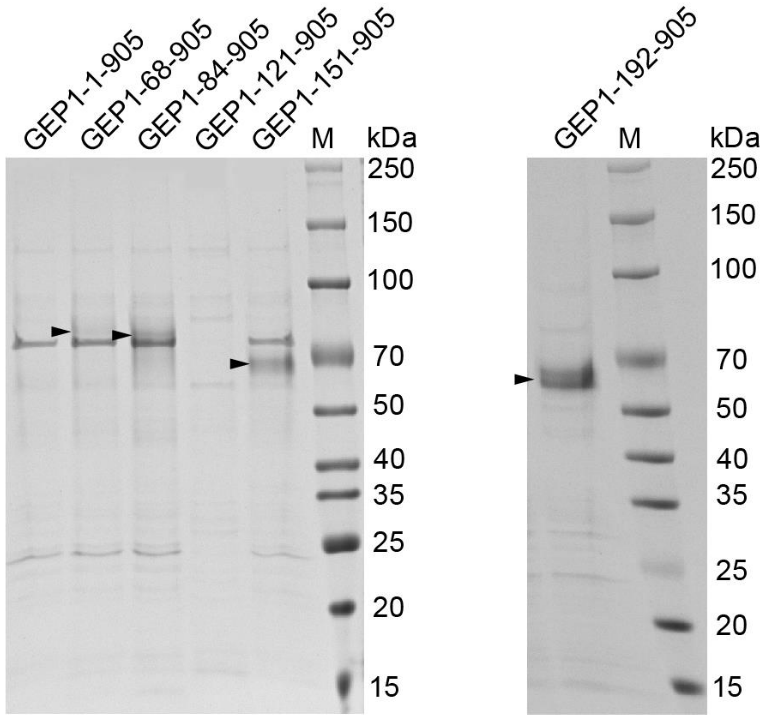


Figure S7. SDS-PAGE analysis of truncations of N-terminal GEP1 by Strep-Tactin beads. Black arrow indicated the location of target band.

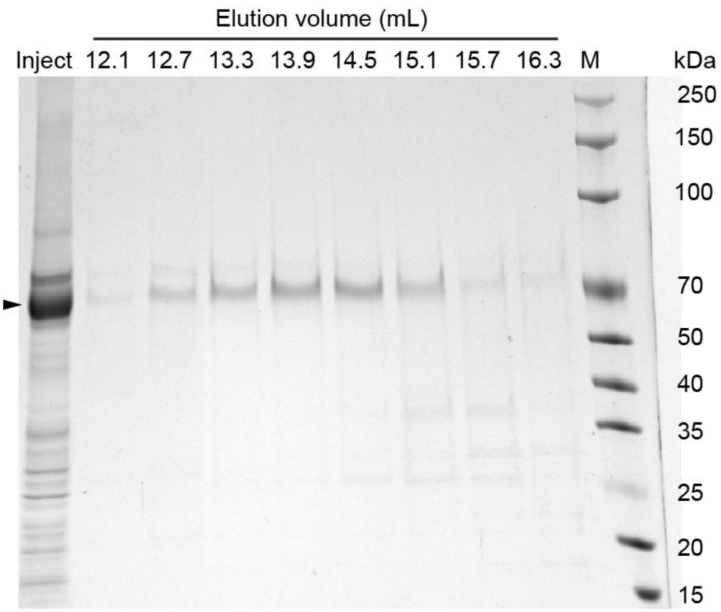


Figure S8. SDS-PAGE analysis of GEP1$^{151\text{-}905}$ purification and size-exclusion chromatography (SEC) fractions in detergent 0.03% DDM. The black triangle represented target band of GEP1$^{151\text{-}905}$.

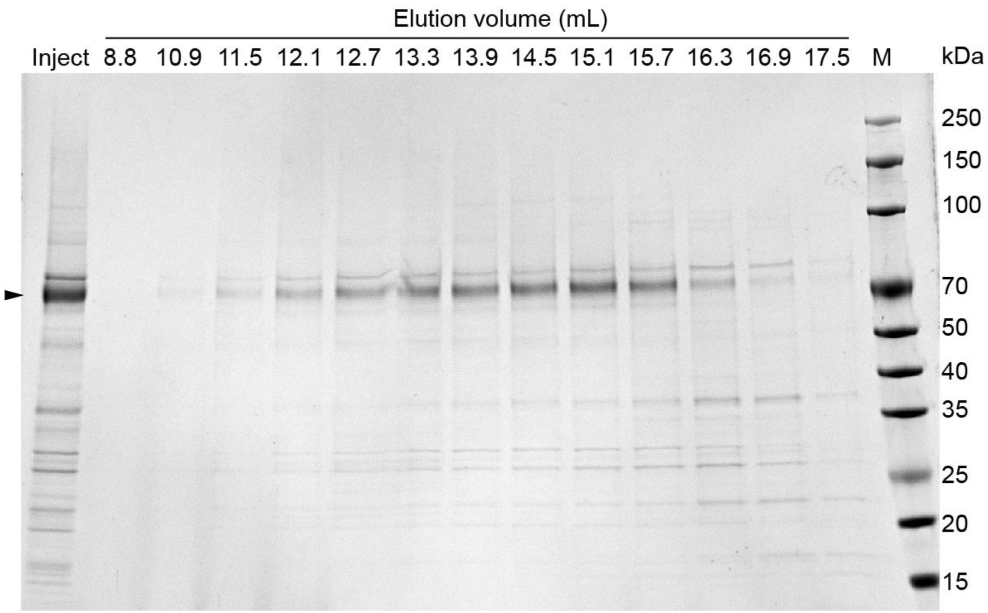


Figure S9. SDS-PAGE analysis of GEP1$^{151\text{-}905}$ purification and SEC fractions in detergent 0.001%/0.00033% LMNG/GDN. The black triangle represented target band of GEP1$^{151\text{-}905}$.

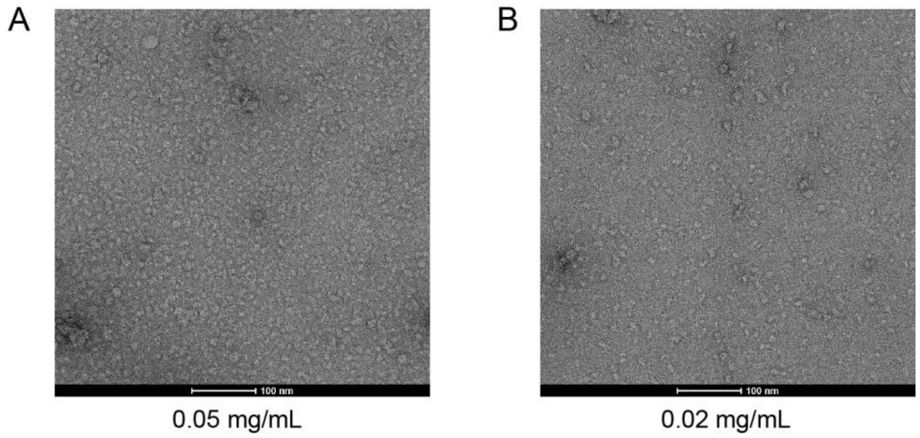


Figure S10. Negative stain electron microcopy of GEP1$^{151\text{-}905}$ in buffer (5 mM Tris-HCl, pH 8.0, 150 mM NaCl, 0.03% DDM). (A) Image of negatively stained GEP1$^{151\text{-}905}$ at concentration of 0.05 mg/mL with 2% uranyl acetate. (B) Image of negatively stained GEP1$^{151\text{-}905}$ at concentration of 0.02 mg/mL with 2% uranyl acetate.

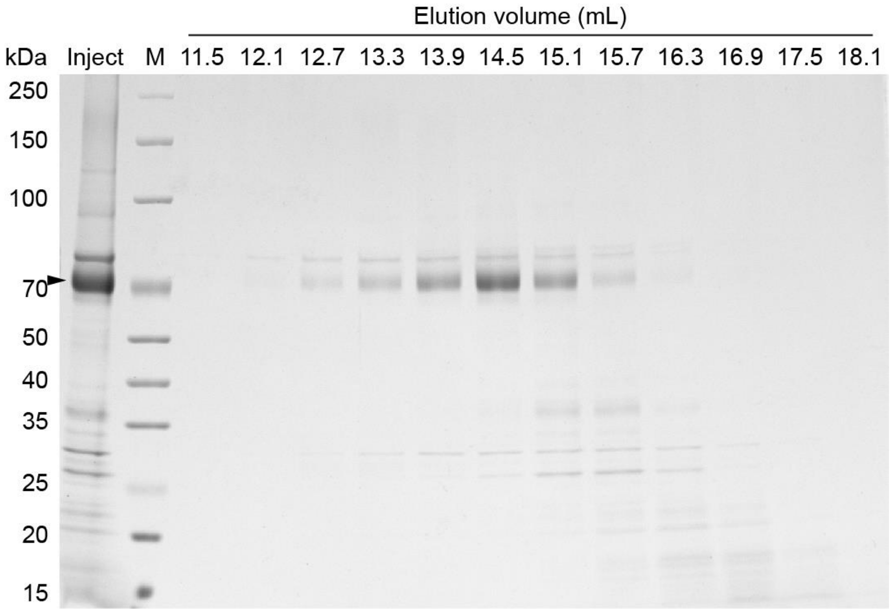


Figure S11. SDS-PAGE analysis of GEP1$^{151\text{-}905}$ purification and SEC fractions trapped in amphipol A8-35. The black triangle represented target band of GEP1$^{151\text{-}905}$.

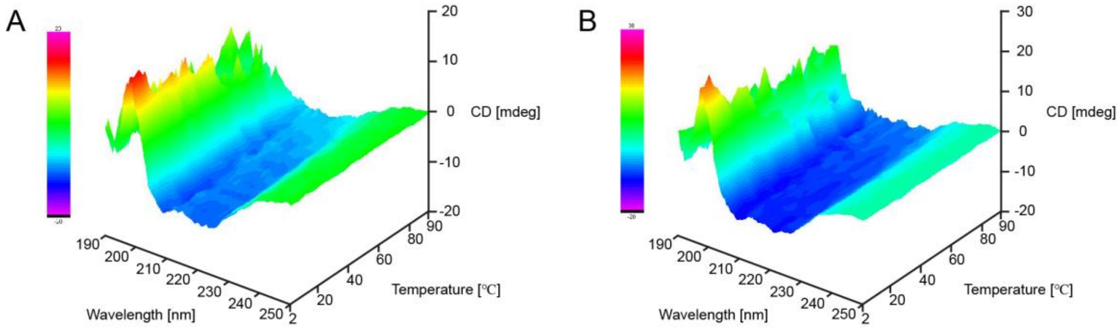


Figure S12. Three-dimensional figure of temperature-dependent CD spectra from 2°C to 92°C, protein GEP1$^{151\text{-}905}$ in buffer pH 7.4 (A) and pH 8.0 (B).

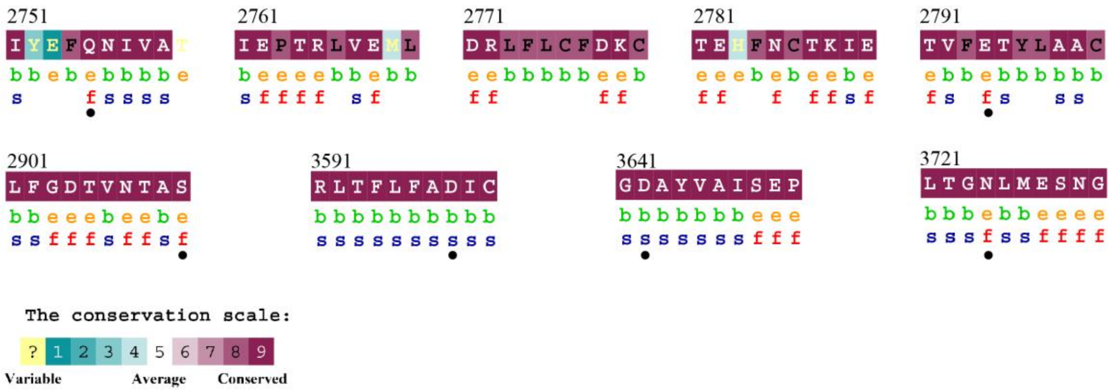

Figure S13. Conservative and functional analysis of several residues in GCα by ConSurf website (https://consurf.tau.ac.il/consurf_index.php). The more conserved residues have a darker red color, residues with low conservation tend to be cyan. Letters under the residues, e: an exposed residue according to the neural-network algorithm; b: a buried residue according to the neural-network algorithm; f: a predicted functional residue (highly conserved and exposed); s: a predicted structural residue (highly conserved and buried).

Table S1. Comparison of proteins structurally similar with GEP1 by DALI server

| No. | PDB | Z-score | Description |
|---|---|---|---|
| 1 | 7LI7 | 26.1 | Sodium-dependent serotonin transporter |
| 2 | 4MME | 25.9 | Transporter |
| 3 | 6VRK | 25.9 | Sodium-dependent serotonin transporter |
| 4 | 5JAG | 25.8 | Transporter |
| 5 | 4MM4 | 25.5 | Transporter |
| 6 | 5JAE | 25.5 | Transporter |
| 7 | 6W2B | 25.4 | Sodium-dependent serotonin transporter |
| 8 | 6W2C | 25.4 | Sodium-dependent serotonin transporter |
| 9 | 4MM9 | 25.3 | Transporter |
| 10 | 7LI8 | 25.2 | Sodium-dependent serotonin transporter |
| 11 | 4XPF | 25.1 | Dopamine transporter-protein |
| 12 | 7MGW | 25.0 | Sodium-dependent serotonin transporter |
| 13 | 4MMB | 24.8 | Transporter |
| 14 | 4MMD | 24.7 | Transporter |
| 15 | 5I73 | 24.7 | Sodium-dependent serotonin transporter |
| 16 | 6M3Z | 24.7 | Sodium-dependent serotonin transporter |
| 17 | 3TT1 | 24.6 | Leucine transporter, LeuT |
| 18 | 4MMC | 24.6 | Transporter |
| 19 | 3TT1 | 24.6 | Leucine transporter, LeuT |
| 20 | 4MMD | 24.6 | Transporter |

Table S2. Comparison of proteins structurally similar with N-terminal helical domain of GEP1 by DALI server

| No. | PDB | Z-score | Description |
|---|---|---|---|
| 1 | 7N7S | 3.3 | Hydroxymethylglutaryl-CoA reductase |
| 2 | 5TTE | 3.1 | E3 ubiquitin-protein ligase ARIH1 |
| 3 | 3P1W | 2.9 | RabGDI protein |
| 4 | 7NPF | 2.9 | AAA family ATPase |
| 5 | 7BY1 | 2.8 | Histone acetyltransferase KAT2A |
| 6 | 3FIG | 2.8 | 2-isopropylmalate synthase |
| 7 | 6IFN | 2.8 | Type III-A CRISPR-associated protein Csm1 |
| 8 | 3T6P | 2.8 | Baculoviral IAP repeat-containing protein 2 |
| 9 | 2MVT | 2.7 | Scoloptoxin SSD609 |
| 10 | 7DN9 | 2.7 | Putative cytoplasmic protein |
| 11 | 5WU1 | 2.6 | Speckle targeted PIP5K1A-regulated poly(A) polymerase |
| 12 | 4M5D | 2.6 | U3 small nucleolar RNA-associated protein 22 |
| 13 | 6HN7 | 2.5 | Redirecting phage packaging protein C (RppC) |
| 14 | 4P17 | 2.5 | RabGAP/TBC protein |
| 15 | 2PX0 | 2.5 | Flagellar biosynthesis protein flhF |
| 16 | 7SHG | 2.5 | Ribofuranosyl transferase |
| 17 | 2KNA | 2.5 | Baculoviral IAP repeat-containing protein 4 |
| 18 | 3EZF | 2.4 | ParA |
| 19 | 4CEJ | 2.4 | ATP-dependent helicase/nuclease subunit A |
| 20 | 6H4C | 2.4 | dUTPase |

Table S3. Primers used to generate constructs with different tags.

| Primer no. | Primer name | Sequence (5'-3') |
|---|---|---|
| 1 | 8H-GEP1-TP_Fwd | GGCGCGGATCCCGGTCCGAAGCGCATATGCATCACCATCACCATC |
| 2 | 8H-GEP1-TP_Rev | CGTCGACGTAGGCCTTTGAATTCCGCTCAGCCTCTGATGGAAAACTCG |
| 3 | 8H-GEP1-mEGFP-TP_Fwd | CGGCGAGTTTTCCATCAGAGGCCTGGAAGTTCTGTTCCAGG |
| 4 | 8H-GEP1-mEGFP-TP_Rev | CGACGTAGGCCTTTGAATTCCGCTCACTTGTACAGCTCGTC |
| 5 | 8H-mEGFP-GEP1-TP_Fwd | CACCATCATCACCACGGATCCATGGTGAGCAAGGGCGAGG |
| 6 | 8H-mEGFP-GEP1-TP_Rev | GCCCCTGGAACAGAACTTCCAGGCCGCTCTTGTACAGCTCG |
| 7 | HA-GEP1-GEP1-TP_Fwd | CGGTCCGAAGCGCATATGAAGACGATCATCGCCCTGAGCTACATCTTC |
| 8 | HA-GEP1-GEP1-TP_Rev | GTCTCCGGCCCGGATCCGGCGAATACCAGGCAGAAGATGTAGCTCAGG |
| 9 | ME-GEP1-GEP1-TP_Fwd | GGTCCGAAGCGCATATGAAATTCTTAGTCAACGTTGCCCTTGTTTTTATGGTCGTAT |
| 10 | ME-GEP1-GEP1-TP_Rev | CTGGTCTCCGGCCCGGATCCATCCGCATAGATGTAAGAAATGTATACGACCATAAAAAC |

Table S4. Primers to generate N-terminal truncations of GEP1.

| Primer no. | Primer name | Sequence (5'-3') |
|---|---|---|
| 11 | GEP1-50-905_Fwd | CATATGGGATCCGGGCCGAGCGTGGGCAGAACCAAG |
| 12 | GEP1-50-905-Rev | CTTGGTTCTGCCCACGCTCGGCCCGGATCCCATATG |
| 13 | GEP1-68-905_Fwd | GGTCCGAAGCGCATATGGGCATCATCACCAAGGAG |
| 14 | GEP1-68-905_Rev | CTCCTTGGTGATGATGCCCATATGCGCTTCGGACC |
| 15 | GEP1-84-905_Fwd | GGTCCGAAGCGCATATGAGCGACGTGAGGAAGATTAG |
| 16 | GEP1-84-905_Rev | CTAATCTTCCTCACGTCGCTCATATGCGCTTCGGACC |
| 17 | GEP1-121-905_Fwd | GGTCCGAAGCGCATATGAACAAGTGTGTCGAGATTAAG |
| 18 | GEP1-121-905_Rev | CTTAATCTCGACACACTTGTTCATATGCGCTTCGGACC |
| 19 | GEP1-151-905_Fwd | CATATGGGATCCGGGCCGTACACCAACCTCTACGAC |
| 20 | GEP1-151-905-Rev | GTCGTAGAGGTTGGTGTACGGCCCGGATCCCATATG |
| 21 | GEP1-192-905_Fwd | CATATGGGATCCGGGCCGTACGATTGCGACAACTTTC |
| 22 | GEP1-192-905-Rev | GAAAGTTGTCGCAATCGTACGGCCCGGATCCCATATG |

Table S5. Primers to generate mutations of GCα-C.

| Primer no. | Primer name | Sequence (5’-3’) |
|---|---|---|
| 23 | Q2755A_Fwd | GTGATATTTATGAATTTGCAAATATAGTTGCAACTATTG |
| 24 | Q2755A-Rev | CAATAGTTGCAACTATATTTGCAAATTCATAAATATCAC |
| 25 | E2794A_Fwd | GAAACAGTTTTTGCAACATATTTAGCTGCTTG |
| 26 | E2794A_Rev | CAAGCAGCTAAATATGTTGCAAAAACTGTTTC |
| 27 | S2910W_Fwd | GATACGGTTAATACTGCTTGGCGAATGAAAACAACTGG |
| 28 | S2910W_Rev | CCAGTTGTTTTCATTCGCCAAGCAGTATTAACCGTATC |
| 29 | D3598A_Fwd | CATTCTTATTTGCTGCAATATGTGGATTTACTTC |
| 30 | D3598A_Rev | GAAGTAAATCCACATATTGCAGCAAATAAGAATG |
| 31 | D3642V_Fwd | TAAATTATGTACAATTGGAGTAGCATATGTTGCAATAAG |
| 32 | D3642V_Rev | CTTATTGCAACATATGCTACTCCAATTGTACATAATTTA |
| 33 | N3724W_Fwd | GATGTATTAACTGGTTTCCTTATGGAAAGTAATGG |
| 34 | N3724W-Rev | CCATTACTTTCCATAAGGAAACCAGTTAATACATC |
